# Unveiling the Pharmacognostic Potential of *Peucedanum ostruthium* (L.) W.D.J. Koch: A Comparative Study of Rhizome and Leaf Essential Oils

**DOI:** 10.3390/plants14132047

**Published:** 2025-07-03

**Authors:** Cristina Danna, Andrea Mainetti, Souda Belaid, Erminia La Camera, Domenico Trombetta, Laura Cornara, Antonella Smeriglio

**Affiliations:** 1 Department of Earth, Environmental and Life Sciences (DISTAV), University of Genova, Corso Europa 26, 16132 Genova, Italy; cristina.danna@edu.unige.it; 2 Forest Botanical Conservation Office, PNGP, Paradisia Alpine Botanic Garden, Valnontey 44, 11012 Aosta, Italy; andrea.mainetti@pngp.it; 3 Department of Chemical, Biological, Pharmaceutical and Environmental Sciences (ChiBioFarAm), University of Messina, Viale Ferdinando Stagno d’Alcontres 31, 98166 Messina, Italy; souda.belaid@unime.it (S.B.); erminia.lacamera@unime.it (E.L.C.); domenico.trombetta@unime.it (D.T.); antonella.smeriglio@unime.it (A.S.)

**Keywords:** plants’ traditional use, masterwort, essential oil, micromorphology, phytochemistry, monoterpenes, sesquiterpenes, antioxidant activity, anti-inflammatory activity, antimicrobial activity

## Abstract

*Peucedanum ostruthium* (L.) W.D.J. Koch (Apiaceae) is a perennial herb native to alpine regions that is renowned in traditional medicine. This study provided a pharmacognostic evaluation, comparing the EOs obtained from its rhizomes and leaves (REO and LEO, respectively). A micromorphological analysis, which was carried out using fluorescence and scanning electron microscopy, revealed terpenoid-rich secretory ducts in both organs. The EOs were extracted by hydrodistillation and characterized by gas chromatography, coupled with flame ionization detection and mass spectrometry (GC-FID and GC-MS), revealing distinct chemical profiles. REO was dominated by monoterpenes (80.08%), especially D-limonene (29.13%), sabinene (19.77%), and α-phellandrene (12.02%), while LEO was sesquiterpene-rich (81.15%), with β-caryophyllene (21.78%), β-selinene (14.09%), and germacrene D (10.43%) as the major compounds. The in vitro assays demonstrated that both EOs exhibit significant antioxidant and anti-inflammatory activities, with LEO consistently outperforming REO across all tests. However, neither EO showed antimicrobial effects against common bacterial or fungal strains. This may have been due to the absence of polar antimicrobial constituents, such as coumarins, which are poorly recovered by hydrodistillation. To fully exploit the therapeutic potential of *P. ostruthium*, especially its antimicrobial properties, future studies should aim to develop integrated formulations combining volatile and non-volatile fractions, preserving the complete plant complex and broadening bioactivity.

## 1. Introduction

*Peucedanum ostruthium* (L.) W.D.J. Koch (syn. *Imperatoria ostruthium* L.) is a perennial rhizomatous herbaceous species belonging to the Apiaceae family. Native to the mountainous regions of central and southern Europe, it thrives in alpine environments, particularly along riverbanks and wetlands, at elevations ranging from 1000 to 2600 m above sea level (a.s.l.). The plant is distinguished by its rhizomatous root system; tripartite leaves (each lobe is trilobed and has serrated margins); erect stems; and white umbelliferous flowers that develop into characteristic fruits known as diachenes [1,2].

Commonly known as masterwort, *P. ostruthium* holds a prominent role in the traditional medicine of the European Alpine regions. Its medicinal use dates back to the 11th century [3] and it is underscored by the variety of its historical names and epithets: *Astrenze/Aschtränze* or *Meisterwurz* (“master”) was used in the Middle Ages and *Imperatoria* (“emperor”) was used during the Renaissance [4,5]. In the 19th century, its rhizome was used in the preparation of *Divinum remedium* [6,7]. Both historical and contemporary ethnobotanical investigations have documented the use of the flowers, leaves, and rhizomes of this species in traditional medicine, with both internal and external applications. Its reported uses span gastrointestinal, respiratory, cardiovascular, dermatological, musculoskeletal, urogenital, endocrine/metabolic, and neurological disorders, as well as conditions related to pregnancy [4,8,9,10,11,12,13,14,15,16,17,18,19,20,21]. These extensive therapeutic applications contribute to its longstanding reputation as a panacea [15,22,23].

The phytochemical profile of *P. ostruthium* is dominated by linear and furanocoumarins—most notably, ostruthin, osthol, and imperatorin—which have been proposed as chemotaxonomic markers in the various extracts examined so far [5,22,23,24,25,26,27,28,29,30,31,32,33,34,35,36,37,38,39,40,41,42,43,44]. These constituents, together with minor polysaccharidic and flavonoid fractions, are consistently associated with marked antioxidant, anti-inflammatory, spasmolytic, and vasodilatory effects in both in vitro and in vivo models [22,23,24,25,26,27,28,29,30,31,32,33,34,35,36,37,38,39,40,41,42,43,44]. Nevertheless, the published data are strongly biased toward hydroalcoholic preparations of the rhizome, whereas chemical and pharmacological information on the essential oils (EOs) remains fragmentary [45,46,47,48]. Crucially, no study has yet compared the EOs distilled from leaves and rhizomes collected from the same botanical population under identical experimental conditions, nor have they linked such comparisons to parallel bioactivity assays [46,47,48]. The present study has filled these gaps by isolating and characterizing both EOs, relating their chemical fingerprints to antioxidant, antibacterial, and anti-inflammatory endpoints, thereby providing new evidence for the therapeutic potential of *P. ostruthium* [22,23,24,25,26,27,28,29,30,31,32,33,34,35,36,37,38,39,40,41,42,43,44,46,47,48]. By adopting this targeted approach, this study offers new insights, which align with and expand upon the current State of the Art. In a previous investigation, hydroalcoholic extracts obtained from both the leaves and rhizomes of *P. ostruthium* were evaluated, providing experimental evidence supporting its traditional medicinal use in the Aosta Valley [15,23]. To further characterize and valorize this emblematic species of traditional Alpine medicine, the present study focused on its essential oils (EOs). Specifically, the rhizomes and leaves were subjected to micromorphological analysis, with an emphasis on their secretory structures. Their EOs were isolated by hydrodistillation, phytochemically characterized, and comparatively assessed. Particular attention was given to their biological activities, including antioxidant, anti-inflammatory, and antibacterial properties.

## 2. Results

### 2.1. Micromorphological Analyses

**Figure 1:**
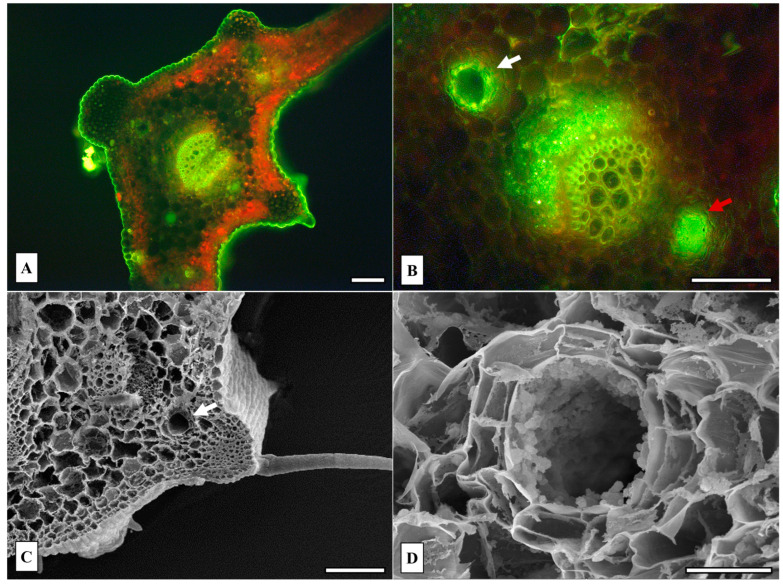
*Peucedanum ostruthium* leaf micromorphology and anatomy. (**A**,**B**) Fluorescence microscopy (FM) of leaf cross sections, H3 filter (420–490 nm). (**A**) Zone of the middle vein. (**B**) Detail showing secretory ducts, positive for Fluorol Yellow 088. The white arrow indicates an empty oil duct, in which the fluorescence of the secretory cells surrounding the duct is visible. The red arrow indicates an oil duct filled with essential oil, showing positive staining. Scanning electron microscopy (SEM) of leaf cross section (**C**) and detail of an oil duct (**D**). Scale bars: 100 μm.

**Figure 2:**
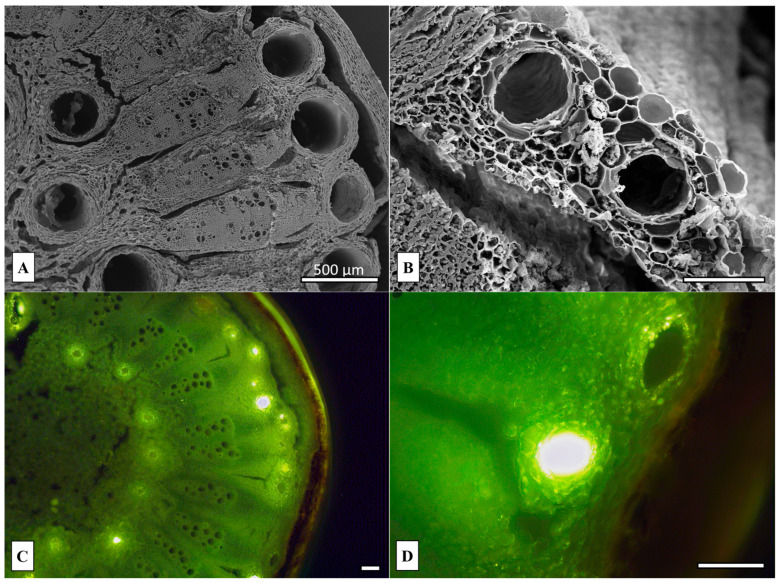
*Peucedanum ostruthium* rhizome micromorphology and anatomy. (**A**,**B**) SEM. (**A**) Cross sections showing the distribution of the ducts. (**B**) Two oil ducts at higher magnification. (**C**,**D**) FM. Cross sections observed with H3 filter (420–490 nm), showing secretory ducts positive for Fluorol Yellow 088. Scale bars: 100 μm, unless otherwise specified.

The micromorphological investigation focused on the secretory structures, particularly the oil ducts present in the leaves and rhizomes of *P. ostruthium*, to document their distribution, morphological features, and histochemical composition (Figure 1 and Figure 2). Light microscopy observations of transverse sections of the leaf, stained with Fluorol Yellow 088, revealed a thick cuticular layer covering both the adaxial and abaxial epidermis (Figure 1A). The leaf exhibited a dorsoventral anatomical organization, and it was characterized by a uniseriate epidermis and typical collenchymatous protrusions associated with the primary and secondary vascular bundles. Additionally, several oil ducts were observed in the midvein region, located above and below the central and lateral vascular bundles (Figure 1A,B). At higher magnification, both the cells lining the oil ducts (Figure 1B, white arrow) and their contents (Figure 1B, red arrow) displayed a positive reaction to Fluorol Yellow 088, indicating the presence of terpenoids.

The use of scanning electron microscopy (SEM) to observe the leaf sections further confirmed the presence of an oil duct situated in the midvein zone (Figure 1C, white arrow) and provided a detailed view of its structural organization, revealing a monolayered secretory epithelium encircling the central lumen (Figure 1D).

The SEM images of the rhizome sections revealed the presence of numerous large oil ducts, arranged in two concentric rings: one encircling the medullary parenchyma and the other surrounding the vascular bundles, located just beneath the cortex (Figure 2A). As observed in the leaves, a higher magnification showed that each oil duct was lined by a monolayered secretory epithelium (Figure 2B). The presence of lipophilic compounds within both the epithelial cells and the oil duct lumina was confirmed by the intense green–yellow fluorescence emitted following staining with Fluorol Yellow 088 (Figure 2C,D), indicating a significant accumulation of terpenoid substances in these tissues.

### 2.2. Phytochemical Analyses

The chemical composition was thoroughly investigated by combining gas chromatography with flame ionization detection (GC-FID) and gas chromatography–mass spectrometry (GC-MS) analyses. This dual-analytical approach ensured both quantitative accuracy and qualitative reliability, allowing for the identification and quantification of volatile constituents with high confidence. As shown in Table 1, a total of 62 compounds were identified across both EOs (of rhizomes and leaves), with substantial differences observed in their relative abundance and chemical classes. The phytochemical profile of rizhome EO (REO) revealed a pronounced dominance of monoterpene hydrocarbons, comprising 80.08% of the total volatile fraction. The most abundant constituents were D-limonene (29.13 ± 0.52%), (±)-sabinene (19.77 ± 0.42%), and α-phellandrene (12.02 ± 0.33%), followed by (-)-α-pinene (6.24 ± 0.08%) and terpinen-4-ol (5.66 ± 0.12%). Other significant compounds included *o*-cymene (4.65 ± 0.11%) and spathulenol (0.72 ± 0.02%).

Sesquiterpene hydrocarbons and oxygenated sesquiterpenes represented a minor portion of the REO, accounting for 6.67% and 1.28%, respectively. The total content of alcohols was 8.80%, while other minor constituents contributed 3.17%. This profile characterizes REO as a monoterpene-rich chemotype, typically linked to notable anti-inflammatory and spasmolytic properties.

The leaf EO (LEO) exhibited a markedly different chemical fingerprint as it was dominated by sesquiterpene hydrocarbons (81.15%) and alcohols (11.39%). The principal constituents were β-caryophyllene (21.78 ± 0.85%), α-caryophyllene (13.95 ± 0.03%), β-selinene (14.09 ± 0.38%), germacrene D (10.43 ± 0.27%), and spathulenol (6.71 ± 0.22%). Other relevant compounds included α-selinene, muurola-4,10(14)-dien-1-β-ol, and caryophyllene oxide.

In contrast to the REO, monoterpene hydrocarbons were poorly represented (3.87%), with D-limonene being the only major monoterpene detected (2.71 ± 0.12%). Oxygenated sesquiterpenes accounted for 3.59%, further confirming the sesquiterpene-rich nature of LEO.

### 2.3. Biological Properties

The antioxidant and anti-inflammatory activities of REO and LEO were evaluated through multiple in vitro assays. All results are expressed as IC_50_ values, representing the concentration required to inhibit 50% of the tested activity, and are presented in mg/mL for EOs and in µg/mL for the reference compounds. Each assay was conducted in triplicate across three independent experiments (*n* = 3), and 95% confidence limits (CLs) were reported (Table 2).

**Figure 3:**
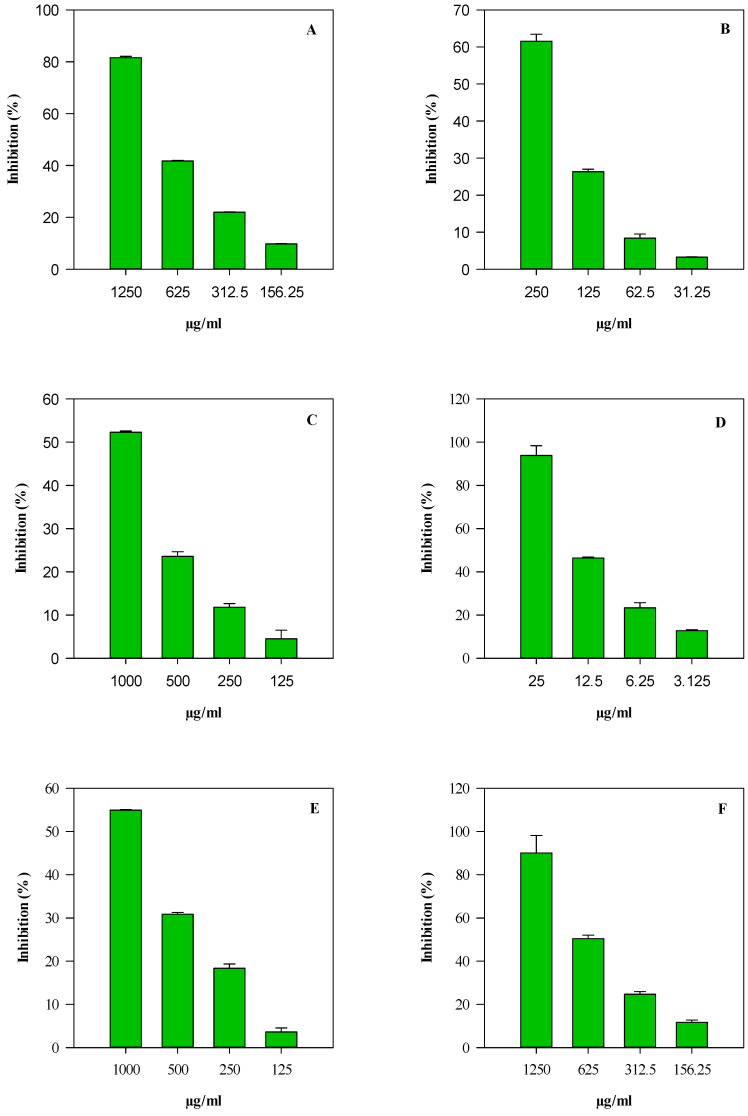
Concentration-dependent antioxidant and anti-inflammatory activity of *Peucedanum ostruthium* leaf essential oil (LEO). Results are expressed as inhibition % and represent the mean ± standard deviation of three independent experiments in triplicate (*n* = 3). (**A**) DPPH; (**B**) FRAP; (**C**) TEAC; (**D**) ORAC; (**E**) albumin denaturation assay (ADA); and (**F**) protease inhibitory activity (PIA).

**Figure 4:**
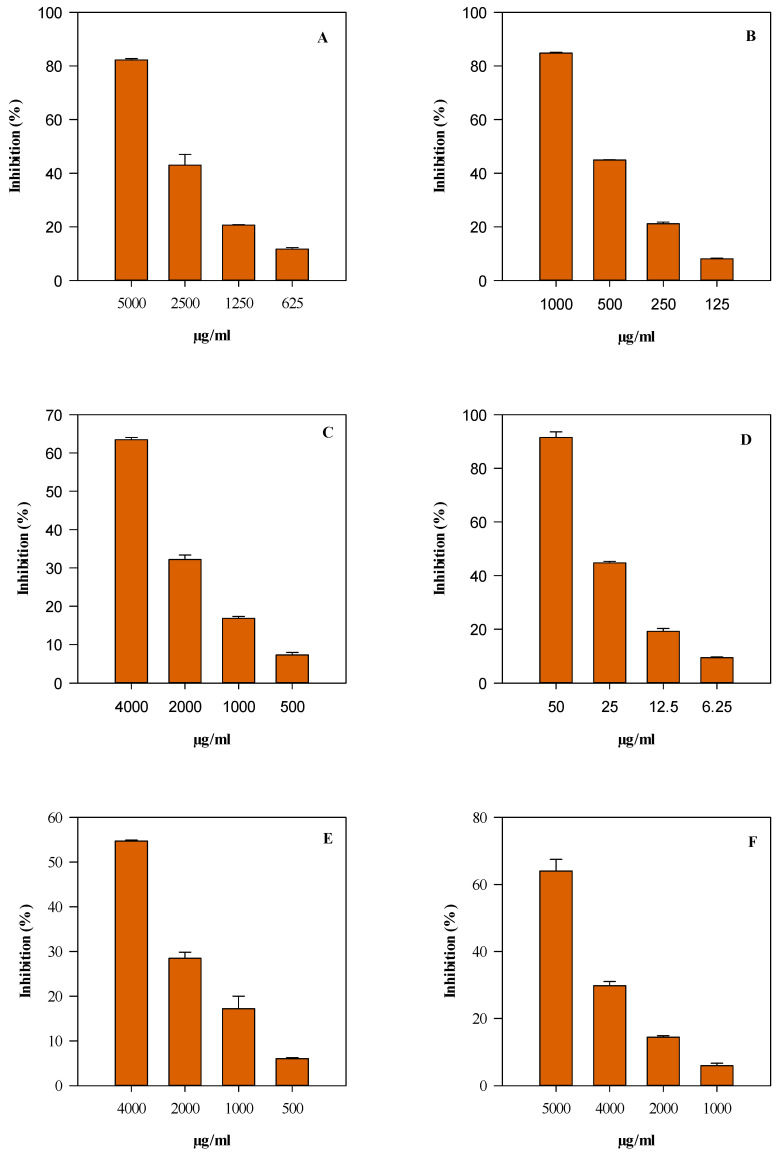
Concentration-dependent antioxidant and anti-inflammatory activity of *Peucedanum ostruthium* rhizome essential oil (REO). Results are expressed as inhibition % and represent the mean ± standard deviation of three independent experiments in triplicate (*n* = 3). (**A**) DPPH; (**B**) FRAP; (**C**) TEAC; (**D**) ORAC; (**E**) albumin denaturation assay (ADA); and (**F**) protease inhibitory activity (PIA).

Both EOs displayed concentration-dependent antioxidant and anti-inflammatory activity (R^2^ ≤ 0.9655) (Figure 3 and Figure 4).

LEO consistently displayed higher and more statistically significant (*p* < 0.001) antioxidant efficacy than REO, except for the ORAC assay. The statistically significant differences observed between the two essential oils (REO and LEO) in the TEAC assay, but not in the ORAC test, can be explained by the distinct reaction mechanisms underlying these methods and the differing phytochemical profiles of the EOs. The TEAC assay is based on a single electron transfer mechanism, measuring the ability of antioxidants to donate electrons to neutralize the ABTS^+•^ radical cation. This method is particularly sensitive to oxygenated sesquiterpenes and other compounds with strong redox potential. LEO—which is characterized by a high content of β-caryophyllene (21.78%), spathulenol (6.71%), and caryophyllene oxide (3.59%)—exhibited a significantly greater electron-donating capacity than REO, which is dominated by less polar monoterpenes, such as D-limonene (29.13%) and sabinene (19.77%). In contrast, the ORAC assay relies on a hydrogen atom transfer mechanism and measures the ability of antioxidants to quench peroxyl radicals by donating hydrogen atoms. This method reflects a broader and more physiologically relevant antioxidant response, which is often influenced by the cumulative and potentially synergistic action of multiple constituents. Therefore, despite their compositional differences, both EOs demonstrated comparable efficacy in the ORAC assay, resulting in no statistically significant difference between them. Similarly, in the DPPH and FRAP assays, LEO confirmed its notable radical scavenging and electron-donating abilities (*p* < 0.001 vs. REO). Although the IC_50_ values observed for the EOs were numerically higher than those reported for pure reference compounds (Table 2), it is important to emphasize that these activities were achieved using a complex phytochemical mixture. EOs are inherently heterogeneous systems that are composed of several volatile compounds, which are often present in variable and synergistic ratios. Therefore, the overall antioxidant performance of these plant complexes must be interpreted as the result of a cumulative and potentially synergistic effect among multiple constituents, rather than the action of a single dominant molecule.

The anti-inflammatory potential of the EOs was investigated using two complementary in vitro cell-free models: the inhibition of albumin denaturation (ADA) and the inhibition of proteolytic enzyme activity (PIA). In both assays, LEO demonstrated a markedly higher activity compared to REO (*p* < 0.001), with IC_50_ values approaching 0.5–0.9 mg/mL. This indicates the significant capacity of LEO to protect protein structures and inhibit the key enzymes involved in the inflammatory cascade. Again, it is critical to consider that these effects derive from the activity of a natural mixture of bioactive terpenes and sesquiterpenes, rather than a purified anti-inflammatory compound. Such plant complexes may exert their effects not only through direct enzyme inhibition or radical scavenging, but also via the modulation of cellular signaling pathways, antioxidant enzyme systems, and membrane stabilization.

Despite their promising antioxidant and anti-inflammatory profiles, neither REO nor LEO showed antimicrobial effects against standard strains of *Staphylococcus aureus*, *Escherichia coli*, *Pseudomonas aeruginosa*, or *Candida albicans*, up to the maximum tested concentration of 2.5 mg/mL.

## 3. Discussion

In a previous study, some micromorphological characteristics of the leaves and rhizomes of *P. ostruthium* were described, including their general anatomical structure [4]. The presence of secretory channels (oil ducts), which are responsible for the production and storage of EOs, represents a typical feature of the Apiaceae family [49]. In the present study, the distribution and histochemical composition of the oil ducts in both the leaves and rhizomes of *P. ostruthium* were further investigated. Notably, the positive response to Fluorol Yellow 088 staining confirmed the presence of lipidic and terpenoid compounds within these structures.

A phytochemical analysis revealed substantial differences between the volatile compositions of LEO and REO. LEO was rich in oxygenated sesquiterpenes—particularly caryophyllene oxide and spathulenol—whereas REO exhibited a monoterpene-dominated profile, with sabinene and 4-terpineol among the most abundant constituents. These findings align only partially with previous reports, such as the study by Garzoli et al. [48], who also observed a sesquiterpene-rich composition in LEO, although their sample contained higher levels of α-humulene and lower levels of spathulenol. These discrepancies may have resulted from environmental factors, the harvesting period, or chemotype variability. Similarly, while our REO was consistent with earlier characterizations of *Peucedanum* rhizome EOs regarding monoterpene predominance, certain constituents reported in the literature, such as α-pinene and germacrene D, were detected in lower amounts or not at all, reinforcing the potential impact of ecological or geographical factors.

Importantly, neither EO contained coumarins, such as osthole or imperatorin, which are compounds frequently cited in non-volatile extracts of *P. ostruthium* for their antimicrobial properties [28,45]. Their absence supports the notion that hydrodistillation does not effectively recover these polar constituents. This is consistent with prior evidence indicating that coumarins, particularly imperatorin and ostruthin, are mainly found in organic or hydroalcoholic extracts and not in volatile fractions [28,39]. The absence of these bioactive coumarins in our EOs may account for the lack of antimicrobial effects observed in this study, despite the plant’s traditional use in the treatment of infections. Indeed, Schinkovitz et al. [28] and Gökay et al. [39] demonstrated the potent antimicrobial activity of *Peucedanum* coumarins that were isolated using organic solvents, but not for their corresponding distilled EOs.

When evaluated for biological activity in this study, both the REO and LEO exhibited significant antioxidant and anti-inflammatory effects, although LEO consistently showed greater efficacy across all in vitro assays. This superior activity can be attributed to its high content of oxygenated sesquiterpenes, which are well-documented for their radical scavenging and anti-inflammatory properties [45,48]. LEO outperformed REO in the DPPH, TEAC, FRAP, and ORAC antioxidant assays. Notably, a statistically significant difference was observed in the TEAC assay but not in the ORAC test, which was likely due to the distinct reaction mechanisms involved: TEAC relies on single electron transfer and is more sensitive to redox-active compounds such as sesquiterpenes, whereas ORAC is based on hydrogen atom transfer and reflects the cumulative and potentially synergistic effects of EO mixtures. Anti-inflammatory activity, which was assessed through protein denaturation and protease inhibition assays, was confirmed for both EOs, although it was more pronounced in the LEO. Although their potency was lower than that of the reference anti-inflammatory agents, these effects are pharmacologically relevant considering the complex multicomponent nature of EOs. Although neither EO contained osthole, the pharmacological importance of coumarins—particularly angular and linear furanocoumarins such as imperatorin and isoimperatorin—as anti-inflammatory agents is well-established in *Peucedanum* species [42]. Despite traditional reports on the use of *P. ostruthium* in the treatment of infectious diseases, neither the LEO nor REO used in this study displayed antimicrobial activity against *Staphylococcus aureus*, *Escherichia coli*, *Pseudomonas aeruginosa*, or *Candida albicans* at concentrations up to 2.5 mg/mL. This observation was in agreement with previous studies on *Peucedanum* EOs, which generally exhibit weak or absent antimicrobial effects unless coumarin derivatives are present [45]. The pharmacological significance of these compounds and their occurrence in polar extracts of *Peucedanum* spp. have been extensively reviewed by Sarkhail [45].

Altogether, these results reinforce the view that sesquiterpene-rich EOs from aerial parts of *P. ostruthium* provide stronger antioxidant and anti-inflammatory effects than monoterpene-rich rhizome EOs. However, the lack of key antimicrobial constituents highlights the need for combined extraction strategies, which preserve both volatile and non-volatile fractions to harness the full therapeutic potential of this species.

Given its superior antioxidant, anti-inflammatory, and wound-healing activities compared to REO, LEO demonstrates promising potential for use in pharmaceutical and dermocosmetic applications. Its bioactive profile suggests that it may serve as a natural ingredient in formulations aimed at managing oxidative stress-related skin conditions, promoting tissue regeneration, or alleviating localized inflammation. In particular, its incorporation into topical preparations, such as gels, creams, or emulsions, could represent a sustainable and functional strategy for the development of plant-based therapeutic products. Further preformulation and pharmacotechnical studies would help to define its feasibility and effectiveness in such contexts.

Although the present study has provided valuable insights into the comparative phytochemical profiles and biological activities of REO and LEO, some limitations should be acknowledged. The experiments were conducted exclusively in vitro, which does not allow for the full extrapolation of the findings to complex biological systems. Moreover, the mechanistic pathways underlying the observed bioactivities, particularly those related to the anti-inflammatory and wound-healing effects, were not investigated at the molecular level. Future studies will be necessary to validate these effects in vivo and to explore the cellular signaling mechanisms involved. Additionally, formulation studies assessing the stability, delivery, and efficacy of these essential oils in relevant biological models are warranted to support potential therapeutic applications.

## 4. Materials and Methods

### 4.1. Plant Material

**Figure 5:**
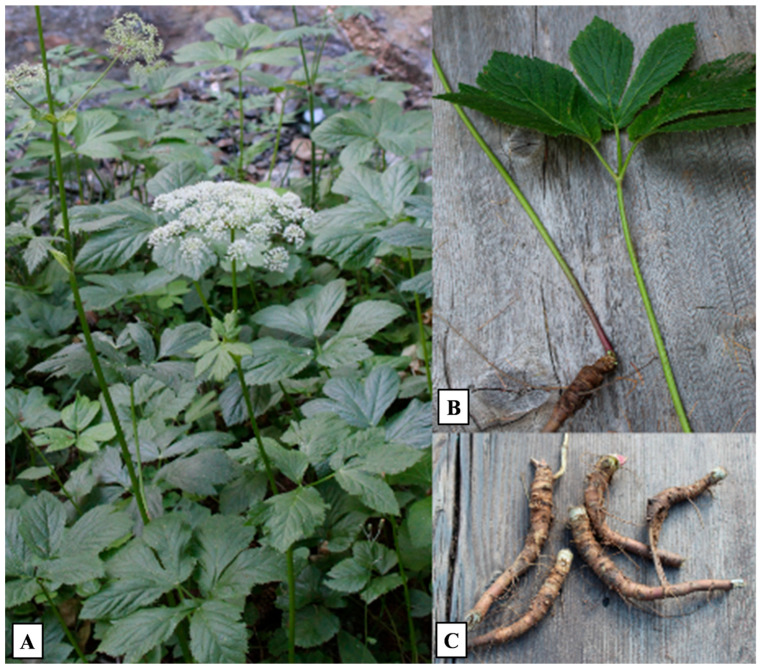
(**A**) Plants of *Peucedanum ostruthium* growing along the riverbank. (**B**,**C**) Higher magnification views of the collected portions (leaves and rhizomes).

The plant material was collected in September and October within the Gran Paradiso National Park, in the locality of Buthier, Cogne, Aosta Valley, Italy (altitude: 1555 m a.s.l.; latitude: 45.603671; longitude: 7.3494468) (Figure 5). A voucher specimen was deposited in the Ethnobotanical Herbarium of the Gran Paradiso National Park, located at the Paradisia Alpine Botanical Garden (Valnontey, Cogne, Aosta, Italy), under the accession code *P.ost. HBPNGP_ETN*.

### 4.2. Micromorphological and Anatomical Investigation

The micromorphological features of both the leaves and rhizomes of *P. ostruthium* were examined using FM and SEM.

For the FM analysis, small portions of fresh leaf and rhizome tissues were manually sectioned with a razor blade. The resulting cross-sections were analyzed using a Leica DM 2000 epifluorescence microscope, equipped with a DFC 320 digital camera (Leica Microsystems, Wetzlar, Germany). The observations were carried out under an H3 filter (excitation 420–490 nm) [49,50,51], following staining with Fluorol Yellow 088, a fluorochrome selective for lipophilic substances, including lipids and terpenoids. The sections were immersed in the staining solution, incubated in the dark for one hour, rinsed with distilled water, and mounted in 50% glycerol prior to observation [52,53,54,55].

For the SEM analysis, the leaf and rhizome samples were cut into small fragments and fixed overnight at 4 °C in a 70% ethanol/Finefix solution (Milestone s.r.l., Bergamo, Italy) [56]. The samples were then dehydrated through a graded ethanol series (70%, 80%, 90%, and 100%), followed by critical point drying using a K850CPD2 system (M Strumenti s.r.l., Rome, Italy). The dried samples were mounted on aluminum stubs with adhesive carbon tabs and sputter-coated with a 20 nm gold layer. Microscopic observations were performed using a Vega3-Tescan LMU scanning electron microscope (TescanUSA Inc., Cranberry Township, PA, USA), operating at 20 kV.

### 4.3. Essential Oil Isolation

Five hundred grams of both fresh leaves and rhizomes of *P. ostruthium* were subjected to hydrodistillation using a Clevenger-type apparatus, following the procedure outlined in the *European Pharmacopoeia* [57]. The distillation process was continued for 3 h, until no further appreciable increase in the EO volume was observed. The resulting pale-yellow EOs, with an extraction yield of 0.52% and 0.76% (*v*/*w*) for the LEO and REO, respectively, were dried overnight over anhydrous sodium sulfate and stored at −20 °C in sealed amber vials under a nitrogen atmosphere until further analysis.

For biological testing, 100 mg of each EO was dissolved in 1 mL of DMSO, producing a 100 mg/mL stock solution. They were properly diluted in methanol or water for the in vitro cell-free experiments.

### 4.4. Phytochemical Characterization

The chemical composition of the EOs was analyzed by both gas chromatography equipped with a flame ionization detector (GC-FID) and gas chromatography equipped with a mass spectrometry detector (GC-MS), using an Agilent 7890A and an Agilent 8890 system, respectively (Agilent Technologies, Santa Clara, CA, USA), according to Smeriglio et al. [58]. Chromatographic separation was achieved using an Agilent J&W HP-5 and an HP-5ms column (30 m × 0.25 mm i.d., 0.25 µm film thickness; Agilent Technologies, Santa Clara, CA, USA), respectively, with helium as the carrier gas, at a constant flow rate of 0.7 mL/min. A 1 µL aliquot of a 10% EO solution in dichloromethane (*v*/*v*) was injected in split mode (50:1), at 250 °C. The oven temperature program was as follows: initial temperature of 60 °C (5 min hold), increased to 100 °C at 3 °C/min, then to 180 °C at 1 °C/min, followed by an increase to 240 °C at 3 °C/min, with a final hold at 240 °C for 5 min. Detector temperatures were set at 280 °C for the FID and 180 °C for the MS. Mass spectrometric detection was performed in electron ionization (EI) mode at 70 eV, with an ion source temperature of 230 °C, and an electron multiplier voltage of 900 V. Mass spectra were recorded in full scan mode, across an m/z range of 40–450. Compound identification was based on multiple criteria: a comparison of linear retention indices (calculated relative to a series of C_7_–C_40_ n-alkanes on the same column); spectral matching against the NIST 20 MS library; an evaluation of the fragmentation patterns reported in the literature [59]; and, where possible, co-injection with authentic reference standards (see Table 1). The quantitative analysis was carried out via GC-FID, and the results were expressed as mean relative peak area percentages ± standard deviation, calculated from three independent experiments, conducted in triplicate (*n* = 3).

### 4.5. Antioxidant and Anti-Inflammatory Assays

The antioxidant and anti-inflammatory activities of *P. ostruthium* EOs were evaluated using a series of in vitro colorimetric and enzymatic assays, each based on distinct reaction mechanisms and test environments and carried out according to Smeriglio et al. [60]. All results represent the mean of three independent experiments performed in triplicate (*n* = 3) and are expressed as a percentage inhibition of the respective activity. The corresponding IC_50_ values, along with 95% CLs, were calculated using the Litchfield and Wilcoxon method, using the PHARM/PCS software (Version 4; MCS Consulting, Wynnewood, PA, USA). All concentrations refer to the final concentration of the EOs or reference compounds in the reaction mixture.

#### 4.5.1. DPPH Radical Scavenging Assay

Briefly, 3.75 µL of REO (625–5000 µg/mL) and LEO (156.25–1250 µg/mL) were added to a freshly prepared 6.3 mM DPPH solution in methanol (1:40, *v*/*v*). The mixture was gently agitated and incubated in the dark for 20 min at room temperature. The absorbance was then measured at 517 nm, using a Varioskan™ LUX multimode microplate reader (Thermo Fisher Scientific, Waltham, MA, USA).

#### 4.5.2. Trolox Equivalent Antioxidant Capacity (TEAC) Assay

The ABTS^+^ radical cation was generated by combining 1.7 mM 2,2′-azino-bis(3-ethylbenzothiazoline-6-sulfonic acid) with 4.3 mM potassium persulfate, followed by incubation in the dark, at room temperature for 12 h. The resulting radical solution was then diluted with ethanol to achieve an absorbance of approximately 0.7 at 734 nm, and used within 4 h of preparation. For the assay, 10 µL of REO (500–4000 µg/mL) and LEO (125–1000 µg/mL) were mixed with the ABTS^+^ reagent (1:20, *v*/*v*) and incubated for 6 min at room temperature. The reduction in absorbance at 734 nm was then measured using the same spectrophotometric microplate reader described in Section 4.5.1.

#### 4.5.3. Ferric-Reducing Antioxidant Power (FRAP) Assay

Briefly, 10 µL of REO (125–1000 µg/mL) and LEO (31.50–250 µg/mL) were added to a freshly prepared, pre-warmed reagent (ratio 1:20, *v*/*v*). The reagent consisted of 300 mM acetate buffer (pH 3.6); 10 mM 2,4,6-tris(2-pyridyl)-s-triazine (TPTZ) dissolved in 40 mM HCl; and 20 mM ferric chloride. The mixture was incubated for 4 min at room temperature in the dark, and absorbance was measured at 593 nm, using the same instrument reported in Section 4.5.1.

#### 4.5.4. Oxygen Radical Absorbance Capacity (ORAC) Assay

Briefly, 20 µL of REO (6.25–50 µg/mL) and LEO (3.125–25 µg/mL) were added to a freshly prepared 117 nM fluorescein solution in phosphate buffer (1:6, *v*/*v*). The mixture was incubated in the dark at 37 °C for 15 min. Subsequently, 60 µL of a 40 mM solution of 2,2′-azobis(2-methylpropionamidine) dihydrochloride (AAPH) was added to initiate the peroxyl-radical-mediated oxidation reaction. Fluorescence readings were taken every 30 s for 90 min, using the same multimode microplate reader described in Section 4.5.1, with excitation and emission wavelengths set at 485 nm and 520 nm.

#### 4.5.5. Bovine Serum Albumin (BSA) Denaturation Assay

The anti-inflammatory potential of REO and LEO was assessed by evaluating their ability to inhibit the heat-induced denaturation of bovine serum albumin (BSA). In summary, a reaction mixture containing 0.4% fatty, acid-free BSA; phosphate-buffered saline (PBS, pH 5.3); and the test samples, REO (500–4000 µg/mL) and LEO (125–1000 µg/mL), was prepared in a 96-well plate, at a volume ratio of 50:10:40 (*v*/*v*/*v*).

The absorbance at 595 nm was measured immediately and then again after 30 min of incubation at 70 °C, using the same microplate reader specified in Section 4.5.1.

#### 4.5.6. Protease Inhibition Assay

In brief, the test samples—REO (1000–5000 µg/mL) and LEO (156.25–1250 µg/mL) —were added to a reaction mixture composed of 10 µg/mL trypsin and 25 mM Tris-HCl buffer (pH 7.5) for a total volume of 400 µL, and mixed in a ratio of 50:3:47 (*v*/*v*/*v*). Following this, 200 µL of 0.8% casein solution was introduced as the enzymatic substrate, and the reaction mixture was incubated at 37 °C for 20 min in a water bath. The enzymatic reaction was then stopped by the addition of 400 µL of 2 M perchloric acid. The resulting suspension was centrifuged at 3500× *g* for 10 min, and the absorbance of the supernatant was measured at 280 nm using a UV-1601 spectrophotometer (Shimadzu, Kyoto, Japan).

### 4.6. Antimicrobial Activity

The antimicrobial efficacy of the EOs was evaluated against standard microbial strains, which were obtained from the in-house culture collection of the University of Messina (Messina, Italy). The tested organisms included *Staphylococcus aureus* ATCC 6538, *Escherichia coli* ATCC 10536, *Pseudomonas aeruginosa* ATCC 9027, and *Candida albicans* ATCC 10231.

The bacterial strains were cultured in Mueller–Hinton broth (MHB, Oxoid, CM0405) and incubated at 37 °C for 24 h, while *C. albicans* was grown in Sabouraud liquid medium (SLM, Oxoid, CM0147) at 30 °C for 48 h.

The minimum inhibitory concentration (MIC) was determined by the broth microdilution method, following the guidelines of the Clinical and Laboratory Standards Institute (CLSI): M100-S22 for bacterial strains [61] and M27-A3 for yeast [62].

### 4.7. Statistical Analysis

The data are presented as the mean ± standard deviation from three independent experiments performed in triplicate (*n* = 3). The statistical analysis was conducted using a one-way analysis of variance (ANOVA), followed by the Student–Newman–Keuls post-hoc test to determine the significant differences. A *p*-value ≤ 0.05 was considered statistically significant. Data processing was performed using SigmaPlot 12.0 software (Systat Software Inc., San Jose, CA, USA).

## 5. Conclusions

This study has reinforced the therapeutic relevance of *P. ostruthium*, primarily due to the antioxidant and anti-inflammatory properties of its leaf EO. While rhizome EO showed moderate activity, it was consistently less effective than leaf EO in all tested assays. The absence of antimicrobial activity in both of the EOs reflected the lack of antimicrobial coumarins, which are typically concentrated in non-volatile plant fractions.

The limitations of the present study included the exclusive focus on essential oils and in vitro models, without in vivo confirmation or the integration of polar phytochemicals.

Future studies should explore the development of an innovative, food-grade extraction technique, which is capable of co-extracting both volatile and non-volatile fractions. Such an approach would enable the formulation of integrated plant complexes, preserving the complete range of bioactive constituents. This strategy is supported by previous findings, which have indicated that hydroalcoholic extracts from the leaves and rhizomes of *P. ostruthium* contain significant amounts of polyphenols, including flavonoids, phenolic acids, and coumarins. Notably, coumarins were particularly abundant in rhizomes, which also demonstrated the most promising wound healing potential, while leaf extracts exhibited superior antioxidant and anti-inflammatory activities. These observations have provided a strong rationale for the combined use of both plant parts in the development of comprehensive formulations with enhanced therapeutic efficacy.

## Figures and Tables

**Table 1:** Chemical composition of *Peucedanum ostruthium* rhizome and leaf essential oil (REO and LEO).

Compound	REO	LEO	KI *^a^*	Identification *^b^*
Octane, 2-methyl	0.08 ± 0.00	−	925	1, 2
β-Thujene	0.97 ± 0.03	−	930	1, 2
α-Pinene	6.24 ± 0.08	−	939	1, 2, 3
Camphene	0.18 ± 0.01	−	954	1, 2, 3
Dehydrosabinene	0.06 ± 0.00	−	962	1, 2
Sabinene	19.77 ± 0.42	−	975	1, 2, 3
β-Pinene	1.00 ± 0.03	−	979	1, 2, 3
β-Myrcene	1.63 ± 0.02	−	990	1, 2, 3
α-Phellandrene	12.02 ± 0.33	−	1002	1, 2, 3
Pseudolimonen	0.30 ± 0.01	−	1004	1, 2
3-Carene	0.22 ± 0.01	−	1008	1, 2, 3
α-Terpinene	0.73 ± 0.02	−	1017	1, 2, 3
o-Cymene	4.65 ± 0.11	−	1026	1, 2, 3
D-limonene	29.13 ± 0.52	2.71 ± 0.12	1029	1, 2, 3
cis-β-Ocimene	0.29 ± 0.01	−	1037	1, 2
trans-β-Ocimene	0.43 ± 0.02	1.16 ± 0.02	1050	1, 2
γ-Terpinen	1.87 ± 0.03	−	1059	1, 2, 3
Terpinolen	0.63 ± 0.02	−	1086	1, 2, 3
o-Cymenene	0.04 ± 0.00	−	1089	1, 2
6-Camphenone	0.40 ± 0.01	−	1096	1, 2
Terpinen-4-ol	5.66 ± 0.12	−	1177	1, 2, 3
trans-Piperitol	0.28 ± 0.01	−	1208	1, 2
cis-Sabinene hydrate	0.50 ± 0.01	−	1221	1, 2, 3
m-Cumenol	1.09 ± 0.03	−	1224	1, 2
(3E,5Z)-1,3,5-Undecatriene	0.19 ± 0.01	−	1230	1, 2
O-Methylthymol	0.12 ± 0.00	−	1235	1, 2
2-Butenoic acid, 2-methyl,4 methylpenthyl ester, (E)	0.68 ± 0.02	−	1265	1, 2
δ-EIemene	0.36 ± 0.01	−	1338	1, 2
β-Bourbonene	−	0.72 ± 0.02	1388	1, 2
β-Elemene	0.35 ± 0.01	0.29 ± 0.01	1390	1, 2, 3
α-Bergamotene	0.16 ± 0.00	1.50 ± 0.03	1412	1, 2
β-Caryophyllene	−	21.78 ± 0.85	1419	1, 2, 3
β-Copaene	−	1.81 ± 0.02	1421	1, 2
Panaginsene	0.17 ± 0.00	−	1425	1, 2
β-Calarene	0.16 ± 0.00	−	1433	1, 2
γ-Elemene	0.43 ± 0.01	−	1436	1, 2
α-Caryophyllene (Humulene)	−	13.95 ± 0.03	1438	1, 2, 3
Aromandendrene	0.46 ± 0.02	1.75 ± 0.02	1441	1, 2
cis-Muurola-4(15),5-diene	−	3.43 ± 0.01	1450	1, 2
trans-Muurola-3,5-diene	−	0.46 ± 0.02	1453	1, 2
γ-Gurjunene	0.19 ± 0.01	−	1477	1, 2
γ-Muurolene	0.09 ± 0.00	−	1479	1, 2
Germacrene D	−	10.43 ± 0.27	1481	1, 2
β-Selinene	1.03 ± 0.02	14.09 ± 0.38	1491	1, 2
α-Selinene	−	6.89 ± 0.23	1498	1, 2, 3
Dihydro-β-agarofuran	1.24 ± 0.02	−	1499	1, 2
Bicylogermacrene	1.67 ± 0.03	−	1500	1, 2
α-Farnesene	−	2.36 ± 0.05	1505	1, 2
cis-Methyl eugenol	0.39 ± 0.01	−	1508	1, 2
δ-Guaiene	0.17 ± 0.00	−	1509	1, 2
Eremophilene	0.94 ± 0.03	−	1510	1, 2
γ-Cadinene	0.11 ± 0.00	−	1513	1, 2
δ-Amorphene	0.38 ± 0.02	−	1515	1, 2
Kessane	1.28 ± 0.03	−	1530	1, 2
α-Cadinene	−	1.70 ± 0.03	1538	1, 2
Spathulenol	0.72 ± 0.02	6.71 ± 0.22	1578	1, 2, 3
Isospathulenol	0.54 ± 0.03	−	1580	1, 2
Caryophyllene oxide	−	3.59 ± 0.11	1583	1, 2, 3
Muurola-4,10(14)-dien-1-β-ol	−	4.68 ± 0.14	1631	1, 2
Total	100	100		
Monoterpene hydrocarbons	80.08	3.87		
Oxygenated monoterpenes	5.94	-		
Sesquiterpene hydrocarbons	6.67	81.15		
Oxygenated sesquiterpenes	2.54	14.98		
Others	4.77	0		

Results are expressed as mean area percentage (%) and ± standard deviation (SD) of three independent determinations in triplicate *(n* = 3): *^a^* Linear retention index on Agilent J&W HP-5MS column, − not detected, and *^b^* Identification method. 1—linear retention index; 2—identification based on the comparison of mass spectra with NIST 20 library; and 3—co-injection with reference standard.

**Table 2:** Antioxidant and anti-inflammatory activity of *Peucedanum ostruthium* rhizome and leaf essential oil (REO and LEO) in comparison with the reference standards.

Test	REO mg/mL	LEO mg/mL	RS ^a^ µg/mL
Trolox equivalent antioxidant capacity (TEAC)	2.98 (2.47–3.59)	1.03 (0.82–1.31) ^§^	4.41 (1.87–10.37) *
Ferric-reducing antioxidant power (FRAP)	0.47 (0.41–0.54)	0.21 (0.17–0.25) ^§^	3.66 (1.63–8.24) *
Oxygen radical absorbance capacity (ORAC)	0.02 (0.01–0.06)	0.008 (0.002–0.01)	0.58 (0.16–2.21) *
2,2-Diphenyl-1-picrylhydrazyl (DPPH)	2.42 (1.29–4.53)	0.61 (0.53–0.72) ^§^	9.44 (4.07–21.93) *
BSA denaturation assay (ADA)	3.98 (3.06–5.16)	0.92 (0.72–1.16) ^§^	20.84 (9.68–44.85) *
Protease inhibitory activity (PIA)	4.91 (1.98–8.21)	0.50 (0.25–1.02) ^§^	27.34 (15.41–48.52) *

Results, which represent the mean of three independent experiments in triplicate (*n* = 3), are expressed as the concentration inhibiting 50% of the oxidant/inflammatory activity (IC_50_), with 95% confidence limits (between brackets). ^a^ RS—reference standard: trolox for FRAP, TEAC, ORAC, and DPPH assay; diclofenac sodium for anti-inflammatory assays (ADA and PIA); * *p* < 0.001 vs. essential oils; and ^§^ *p* < 0.001 vs. REO.

## Data Availability

Data are contained within the article.
